# *Fagus sylvatica* seedlings show provenance differentiation rather than adaptation to soil in a transplant experiment

**DOI:** 10.1186/s12898-018-0197-5

**Published:** 2018-10-03

**Authors:** R. D. Manzanedo, F. R. Schanz, M. Fischer, E. Allan

**Affiliations:** Institute of Plant Sciences, University of Bern, Altenbergrain 21, 3013 Bern, Switzerland

**Keywords:** Ectomycorrhizae, Plant-fungal interactions, Drought resistance, Genetic adaptation, Reciprocal transplant, Soil

## Abstract

**Background:**

Understanding and predicting the response of tree populations to climate change requires understanding the pattern and scale of their adaptation. Climate is often considered the major driver of local adaptation but, although biotic factors such as soil pathogens or mutualists could be as important, their role has typically been neglected. Biotic drivers might also interact with climate to affect performance and mycorrhizae, in particular, are likely to play a key role in determining drought resistance, which is important in the context of adaptation to future environmental change. To address these questions, we performed a fully reciprocal soil–plant transplant experiment using *Fagus sylvatica* seedlings and soils from three regions in Germany. To separate the biotic and abiotic effects of inoculation, half of the plants were inoculated with natural soil from the different origins, while the rest were grown on sterilized substrate. We also imposed a drought stress treatment to test for interactions between soil biota and climate. After 1 year of growth, we measured aboveground biomass of all seedlings, and quantified mycorrhizal colonization for a subset of the seedlings, which included all soil–plant combinations, to disentangle the effect of mycorrhiza from other agents.

**Results:**

We found that plant origin had the strongest effect on plant performance, but this interacted with soil origin. In general, trees showed a slight tendency to produce less aboveground biomass on local soils, suggesting soil antagonists could be causing trees to be maladapted to their local soils. Consistently, we found lower mycorrhizal colonization rate under local soil conditions. Across all soils, seedlings from low elevations produced more annual biomass than middle (+ 290%) and high (+ 97%) elevations. Interestingly, mycorrhizal colonization increased with drought in the two provenances that showed higher drought tolerance, which supports previous results showing that mycorrhizae can increase drought resistance.

**Conclusions:**

Our findings suggest that soil communities play a role in affecting early performance of temperate trees, although this role may be smaller than that of seed origin. Also, other effects, such as the positive response to generalists or negative interactions with soil biota may be as important as the highly specialized mycorrhizal associations.

**Electronic supplementary material:**

The online version of this article (10.1186/s12898-018-0197-5) contains supplementary material, which is available to authorized users.

## Background

Adapting to climate change will be a great challenge for many ecosystems as climate patterns alter and extreme events, such as droughts, become more frequent [1]. One important factor that will likely influence the environmental response of natural populations is the scale and drivers of adaptation (e.g. [2]). While the existing literature shows that a majority of populations exhibit patterns of local adaptation [3, 4], the drivers of these patterns are less clear. Climatic conditions are the most frequently invoked factor to explain adaptive divergence, but climate may explain only a small part of the local advantage [4, 5]. Adaptation to local biotic factors, such as soil biota, may also be important but has been less frequently considered.

Soil biota play a key role in the functioning of ecosystems, influencing plant performance, community structure and ecosystem dynamics [6, 7]. Soil communities are also highly diverse and variable at small spatial scales across the landscape [8]. It is therefore likely that plants can gain an important advantage by genetically adapting to their local soil community [9]. For instance, Pickles et al. [10] showed that most populations of a widespread tree species, *Pseudotsuga menziesii,* are locally adapted across the species range and suggested that soil fungi were the main drivers of this pattern. Because of their longevity and large geographic range, tree species encounter a range of different soil communities and there is therefore a large potential for them to specialize to their local soil biota. In some cases, single individuals have even been shown to perform better with their very local soil community [11]. In contrast, there have been reports of partial [12–14] or general maladaptation to soil [15], suggesting that other factors and interactions determine whether trees locally adapt to their soils. These cases of maladaptation can be caused by the faster generation time of antagonistic soil microbes, which may mean they are able to rapidly overcome the hosts’ defences [16]. As stressed by Rúa et al. [9], in a recent meta-analysis on the extent of plant local adaptation to soil, while there are strong reasons to suspect that soil biota play a key role in plant local adaptation, it is important to investigate how strongly different populations are adapted to their local soil community, and whether this interaction is dominated by mutualistic or parasitic associations.

The effects of soil biota on trees, and their role in generating local adaptation, can also determine how plants respond to global change, particularly to increasing drought [17]. An increase in drought frequency and severity with climate change [1] is likely to cause increased plant stress and may even result in episodes of mass tree mortality [18, 19]. This could be offset by beneficial soil microbes, which may confer drought resistance: for instance, substantial reductions in soil moisture (more than 7%) have been shown to differentially affect shoot biomass, photosynthetic rate and leaf nitrogen of mycorrhizal and non-mycorrhizal plants [20]. Specific combinations of mycorrhizal and host genotypes have also been shown to completely alter the host’s drought tolerance and, potentially, its response to future environmental change [21]. It has even been suggested that increasing temperatures may increase the frequency of positive mycorrhizae-plant interactions [22], which might in turn enhance differences between populations if mycorrhizae are drivers of local adaptation. An increase in aridity can therefore indirectly affect trees by influencing their interactions with soil biota [23]. However, more experiments are needed to confirm these effects and to increase our understanding of the consequences of climate change for plant-soil pathogen interactions (discussed in [24, 25]). The lack of consistent patterns in the interactions between mycorrhizal fungi, plants and global change drivers ([17, 26, 27]), calls for more controlled experiments that test the interacting effects of environmental stress and specialized soil biota [23].

To predict and manage climate change impacts, it is imperative to understand how economically and ecologically important species will respond to future changes. In Europe, where a few tree species dominate the landscape, their ability to adapt to new climatic conditions greatly influences the functioning of large natural ecosystems. European Beech (*Fagus sylvatica*, hereafter ‘*Fagus*’) is one of the most common tree species in Europe. It is also one of the most extensively studied species, due to its wide distribution and economic importance [28]. *Fagus* populations are almost continuous in the centre of their range, in central Europe. As a consequence, populations are connected by high gene flow (reviewed in [29]) suggesting that between-population genetic differentiation may be low. At the same time, the populations encounter a large range of environmental conditions, including gradients in climate, topography, and potentially in biotic interactions. This, and the reported, high within-population genetic variability [30], suggests that *Fagus* populations have a large potential for small-scale genetic differentiation [31]. In fact, adaptation to very local conditions has already been reported for *Fagus* in marginal and central parts of the range [32], as well as along elevational gradients [33]. Although there is a large body of research on the adaptation of *Fagus* to climate, the extent to which it might be adapted to biotic factors and how these might interact with changing climatic conditions is still largely unexplored.

Here, we examined the interactive effects of local mycorrhizae, tree provenance and drought on early tree growth in a reciprocal soil–plant transplant experiment. This experiment was carried out under common garden conditions with soil and seedlings of *Fagus sylvatica* from three regions in Germany that are located at three distinct elevations. To assess the interacting effects of soil communities (especially ectomycorrhizal fungi) and drought on tree performance, we applied fully factorial drought and soil inoculation treatments to our seedlings. Specifically, we addressed the following questions: (i) do *Fagus* seedlings produce more aboveground biomass when growing in soil inoculated with biota from their local origin? (ii) Are mycorrhizal associations more frequent in local soil conditions? (iii) Does drought modify plant–soil biota interactions? And (iv) does mycorrhizal colonisation affect plant tolerance to drought?

## Methods

### Biodiversity Exploratories and the tree provenances

Our experiment was conducted within the context of the German Biodiversity Exploratories. They are an interdisciplinary and long-term scientific program that studies the effects of land-use intensity and biodiversity on ecosystem functioning in German forests and grasslands. Plots are located in three regions: in the North-East, Center, and South-West of Germany: Schorfheide-Chorin (hereafter ‘Low’), Hainich-Dün (hereafter ‘Middle’), and Schwäbische Alb (hereafter ‘High’). From North to South, the elevations of our study regions are: E_Low_ = 3–140 m, E_Middle_ = 285–550 m, and E_High_ = 460–860 m a.s.l. [34]. As a consequence, temperature and precipitation between regions do not vary as would be expected solely from their latitude. We therefore refer to the soil and plant origins by their elevations, rather than by their latitudinal positions, as this more clearly indicates the climatic differences between them. Populations at higher elevations (southern) actually experience higher precipitation (both annually and during summer) and lower temperatures than those located at middle and low elevations (central and northern latitudes respectively), and thus experience higher water availability at several soil depths (see Table 1). The most drought stressed region is therefore the low elevation one, followed by the middle and then high elevation regions.

**Table 1:** Mean climatic parameters for each Biodiversity Exploratory region, as measured directly on the monocultural *Fagus sylvatica* plots from 2009 to 2016

Exploratory site	Code	Summer P (mm)	Summer T (°C)	Annual P (mm)	Annual T (°C)	Soil moisture at 10 cm (%)	Soil moisture at 20 cm (%)
Schorfheide-Chorin	Low	177	16.96	623	8.57	13.67	10.49
Hainich-Dün	Middle	169	15.40	512	7.67	26.19	25.62
Schwäbische Alb	High	273	15.27	808	7.45	32.49	28.21
Bern	CG	358	17.00	986	8.60	–	–

Annual values for precipitation and temperature (*Annual P, Annual T*) express whole-year average across plots, while seasonal values (*Summer P and Summer T*) are June–August averages. *Soil moisture 10* cm and *Soil moisture 20* cm are June to August mean percentages of volumetric water content at the specified depth. The temperature and precipitation conditions for the location of the common garden experiment (Bern, CG) are also shown

In our experiment we used the three Exploratory regions as tree provenances (i.e. distinctive seed origins). Between February and April 2014, we collected 2–3 year old naturally regenerated *Fagus* seedlings, together with surrounding soil samples, from each of the three Biodiversity Exploratory regions. We chose to work with naturally regenerated seedlings, so that we could study effects of soil and drought on older seedlings, which have already passed the environmental filters imposed during the germination and establishment stages. This would not have been possible if we had established the seedlings from seeds germinated under controlled conditions. We took the soil and seedlings from mature monocultural *Fagus* stands, located near to the site of seedling collection in each region (see [34]). We collected approx. 4 L of top soil, which was then sieved to remove big particles, roots and other organic material and mixed thoroughly before being used for the inoculations. We also added to this the soil obtained from gently shaking the roots of the seedlings, as this rhizosphere soil may be more likely to contain mycorrhizal spores. In total, we collected 450 seedlings from the three regions. We brought them to the Botanical Garden of the University of Bern (Switzerland) to conduct the experiment. The seedlings from the low and high elevation regions were transported by car immediately after collection while those from middle elevations were sent by mail.

### Experimental design

All phases of the experiment took place in a location independent of the three provenance origins, in the Botanical Garden of Bern, Switzerland (46°57′07″ N–7°26′43″E, 501 m a.s.l.). Bern has a temperate climate, with mean annual temperatures of 8.6 °C and mean annual precipitation of 986 mm (see Table 1). This means Bern is warmer and wetter than any of the source regions, meaning no provenance should be best adapted to the local climate. This allowed us to focus on the effects of the soil community and the experimentally imposed drought. The experiment followed a complete factorial design with 36 different treatment combinations (3 soil origins × 3 plant origins × 2 inoculation treatments × 2 drought treatments). We randomly assigned the seedlings to one of 25 blocks, each one including all possible combinations of soil, plant, and inoculation. Plants were randomly positioned within each block. Before planting, we washed the root systems of the seedlings to remove all adhering soil and fungal material. We did not cut off the potentially mycorrhized root tips of the seedlings, as this could have seriously damaged their root system and compromised their survival. We used a standard seedling substrate, which was a blend of white and black sphagnum peat buffered with coco fibre (Klasmann-Deilmann GmbH, Geeste, Germany) that had previously been sterilized by autoclaving. This soil mix is commonly used in greenhouses and forest nurseries and it has previously been used in studies looking at mycorrhizae, which have shown that it is a suitable substrate for ectomycorrhizae and good colonisation has been observed on seedlings grown in this soil [35]. All seedlings were planted in 13 × 13 × 13 cm square pots. We inoculated the pots with 30 mL of natural soil, thoroughly mixed with the standard substrate before planting (see [36]). We preferred this inoculation method over a complete soil reciprocal transplantation to minimize the effect of abiotic differences between soils. Half of the seedlings were inoculated with natural soil from the three origins and, to further separate biotic from abiotic effects of soil addition, the seedlings in the ‘not inoculated’ treatment received 30 mL of sterilized soil from the corresponding soil origin. The sterilization of the inoculum liberated the organic nitrogen in the soil (see Additional file 1: Fig. S1, calculated as total soil nitrogen). It is difficult to say whether this is a permanent release, but we consider this effect to be negligible as the amount of inoculum was small relative to the volume of standard substrate and the standard substrate was fairly rich in nitrogen (Additional file 1: Fig. S1). In any case, in order to reduce the potential effect of nutrient liberation due to sterilization, 2 months after establishing the experiment, we fertilized each pot with 3 g of a slow-release fertilizer rich in nitrogen, phosphorus and potassium (Tardit Top, Hauert Günther Düngerwerke GmbH, Nuremberg, Germany). It possible, although unlikely, that this low level of fertilization affected mycorrhizal colonization (see e.g. [37]) and community structure. However, as the levels of added nutrient were low, we expect this effect to be small. To prevent cross-contamination between pots via watering, we placed them on a raised wooden structure.

During establishment all plants were watered for 1 min per block every other day, which was sufficient to maintain soil humidity. Following this, on July 27th 2015, we moved all plants to an open-sided greenhouse, adjacent to the common garden. This was done in order to control water input, since 12 of the blocks were randomly assigned to a 6 week drought treatment, lasting until 6th September. Other than preventing any precipitation reaching the seedlings, the conditions inside the greenhouse are expected to be similar to those outside. The drought treatment simulated a 50% reduction of water during a short-to-medium length drought, which, according to most climate change scenarios, is likely to become much more frequent in the future [1]. We chose a slight drought treatment over a moderate or extreme one as we aimed to study the consequences of a realistic reduction in water availability for the seedlings in the near future [1]. Consequently, the response of plant-soil interactions to severe or extreme drought conditions, though an interesting question, lay outside of the scope of this paper and should be addressed in future work. During the treatment, ‘Drought’ blocks were watered every 4 days for 1 min per block, while ‘control’ blocks were watered every other day. The drought plants showed signs of stress, with strong leaf wilting and slight discoloration, but no leaf loss was reported. Due to very high temperatures inside the greenhouse from August 5th to August 15th, we decided to reduce the intensity of drought by watering control seedlings daily and drought seedlings every other day. Afterwards, the drought treatment was resumed as before. At the end of the treatment the plants were moved back to their original location, outside of the greenhouse, until the end of the experiment.

### Measurements

At the beginning of the experiment, we measured plant height to account for initial size differences and to control for potential maternal or storage effects [38]. In November and December 2015, after the seedlings had shed their leaves and entered winter dormancy, we collected the biomass of the seedlings, above the root collar. The samples were air dried in the oven at 70 °C for 3 days and then quickly weighed with a precision balance before they were rehydrated. We removed occasional dry leaves still attached to the seedlings before weighing. Final aboveground biomass values were corrected for initial height so that we measured aboveground biomass growth in the experiment.

We also quantified ectomycorrhizal fungi on the root system of a subset of the seedlings. We selected 6 blocks of 18 seedlings each (3 from the drought and 3 from the control treatment, 108 individuals in total) to quantify and identify their mycorrhizal associations. We used the staining protocol for mycorrhizal quantification from Vierheilig et al. [39] after adjusting the heating times and KOH concentrations to maximize mycorrhizal visibility in our samples. We took 10 segments of thin root tips per tree, cleaned them with water and submerged them at 90 °C in a 10% KOH solution for 10 min. After rinsing with distilled water we stained them with a 10% ink-vinegar solution for 2 min at boiling temperature, and again cleaned them with distilled water. We kept the samples in slightly acidified water over night to remove any excess stain. The stained root fragments were inspected under a 16 × − 64 × stereoscopic magnifier (Leica AG, Wetzlar, Germany) as the colonized roots are clearly visible following staining. We measured the number of total and mycorrhizal tips for all root fragments and averaged them to produce a single estimate of mycorrhizal colonization per seedling. Although the staining method is more often used in arbuscular mycorrhizal associations, it is also sometimes used to characterize ectomycorrhizae (e.g. [40]), because it allows researchers to visualize the Hartig net under the stereoscope, and thus provide a better estimate of the abundance of active mycorrhizae [41]. However, we acknowledge that this may come at a cost of underestimating the colonization rate of very superficial fungi species, which may have been damaged by the clearing method. Similarly, since clearing and staining may affect mycorrhizal morphotype characteristics, we did not assess the diversity and species composition of the mycorrhizal communities in our samples.

Unfortunately, we have no measurements of root biomass, which would have complemented the measures of aboveground biomass and could have clarified the observed patterns. Two reasons played a role in our decision not to measure belowground biomass. First, it is not possible to measure belowground biomass non-destructively and root length-biomass relationships are not reliable for tree seedlings [42]. We therefore felt that we could not measure root biomass at the end of the experiment because such a measure would have been too strongly affected by any difference in initial mass and, unlike for aboveground biomass, we could not correct for these initial differences in root biomass. Second, the few studies that explored the relationship between above- and below- ground biomass through the lifecycle of trees have reported a consistent and strong correlation between them (e.g. [43]). Therefore, although we only measured aboveground biomass growth, we hope that our results capture the general patterns of seedling performance and that the measured change in aboveground biomass should correlate with the change in belowground biomass. However, it is certainly possible that we would have found clearer responses if we had measured root biomass production during the experiment.

### Statistical analysis

We used linear mixed models (*lme*) to test the effects of plant and soil origin on aboveground biomass (*AGB*). We included block as a random factor to account for potential spatial variability within the experimental site. The full model for AGB included all possible interactions between variables:

$$\begin{aligned} lme \, (log\left( {AGB} \right)\,\sim \,soil.origin \, * \, plant.origin \, \quad * \, drought \, * \, inoculum\, + \,height.0, \, random \hfill \\ \, = \,\sim 1|block, \, method = \hbox{''}ML\hbox{''}) \hfill \\ \end{aligned}$$

Aboveground biomass was log-transformed to meet normality and heteroscedasticity model assumptions. Initial plant height was included as a covariate. Consequently, biomass estimates presented here are standardized by initial height, so that they represent changes in biomass during the experiment (see [38]).

To analyse mycorrhizal colonization (*mycoperc*) we used generalized linear models (*glm*). We treated block as a fixed effect, which allowed us to fit glms instead of mixed models. The full model was then defined as:

$$\begin{aligned} glm \, (mycoperc\,\sim \,soil.origin \, * \, plant.origin \, \quad * \, drought \, * \, inoculum\, + \,block, \, family \\ \, & = \,quasibinomial) \\ \end{aligned}$$

We used a quasibinomial distribution to reduce the overdispersion problems we encountered when using a simple binomial [44]. In all cases, we did model selection by backward deletion, based on likelihood ratio tests and the differences in Akaike’s Information Criterion (ΔAIC of more than 2 AIC units was considered threshold for variable selection) between models to find the most plausible model [44]. All statistical analyses were performed with the R software [45] using the ‘nlme’ to fit linear mixed models [46] and ‘MuMIn’ package to calculate R^2^ values for the mixed models [47].

## Results

**Fig. 1:**
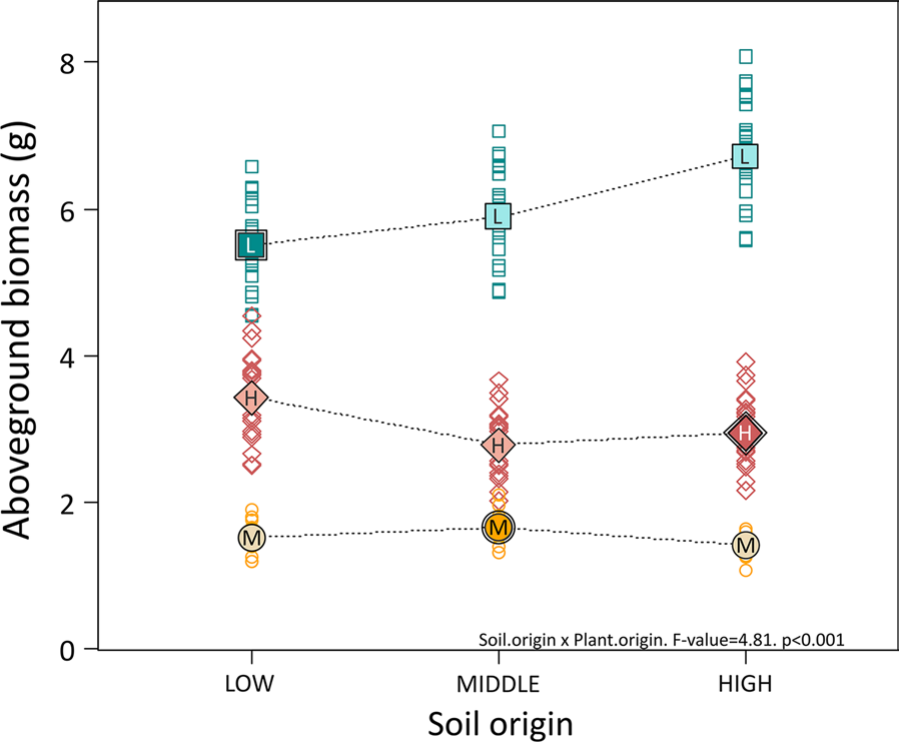
Dried aboveground biomass by soil origin and plant origin in the common garden experiment. Open symbols are model predictions after standardizing by initial size. Mean values are displayed as filled symbols with letter code per region. Means of local combinations are displayed as darker colour and with double symbol border. Regions names coded as in Table 1. F-value and significance of the interaction are also displayed

Seedling performance was affected both by plant and soil origin (Fig. 1). However, the comparison between provenances showed that, after correcting for initial conditions, seedlings from the low elevation provenances produced most biomass and this was consistent in all soils. Low elevation seedlings produced on average 4.47 g more biomass (+290%) than those from middle elevations; and 2.96 g more (+97%) than the high elevation provenances (Fig. 1, Additional file 1: Table S3). This strong hierarchy suggests clear genetic differentiation between provenances. However, maternal effects and storage could also play a role in driving such patterns and, despite our efforts to minimize them by standardizing by initial size, we cannot completely rule out this possibility.

Seedlings from both high and low elevation provenances performed worse in local soil than they did in foreign soil, while seedlings from middle elevations performed slightly better in their own soil (plant origin by soil origin interaction, Table 2, Fig. 1). In the high and low provenances, this may suggest either local maladaptation to mutualists such as mycorrhizae, or stronger negative soil feedback from local pathogens. However, the lack of a significant interaction between inoculation (live vs. sterile) and soil origin (p = 0.10, Table 2), suggests that this response may have been at least partially caused by abiotic differences. The chemical characterization of the soils showed that sterilization strongly modified the concentration of nitrate, phosphate and ammonia (Additional file 1: Fig. S1). Soil pH, on the other hand, remained largely unaffected by sterilization, as expected (Additional file 1: Table S2). Middle elevation soils had neutral pH, while the high and low ones were slightly acidic. This was also true for the standard substrate, which may contribute to explain the poorer performance of middle elevation seedlings in our experiment.

**Table 2:** Model selection and levels of significance in the linear mixed model analysing seedling aboveground biomass

Aboveground biomass					
Explanatory variable	numDF	F-value	p-value		ΔAIC
***Intercept***		1668.74	< 0.0001	***	
***Height.0***	1	165.20	< 0.0001	***	
***Drought***	1	3.44	0.08	n.s.	
***Soil.origin***	2	2.84	0.06	n.s.	
***Plant.origin***	2	254.28	< 0.0001	***	
***Drought : plant.origin***	2	3.78	0.02	*	
***Soil.origin : plant.origin***	4	4.81	0.0009	**	
Inoculum	1		0.24	n.s.	− 0.61
Soil.origin : inoculum	2		0.10	n.s.	+ 0.60
Drought : soil.origin	2		0.36	n.s.	− 1.96
Drought : inoculum	1		0.65	n.s.	− 1.79
Drought : soil.origin : inoculum	2		0.45	n.s.	− 2.42
Drought : soil.origin : plant.origin	4		0.80	n.s.	− 6.35
Plant.origin : inoculum	2		0.91	n.s.	− 3.81
Drought : plant.origin : inoculum	2		0.80	n.s.	− 3.54
Soil.origin : plant.origin : inoculum	4		0.80	n.s.	− 6.38
Drought : soil.origin : plant.origin : inoculum	4		0.86	n.s.	− 6.69

Variables are shown in reverse order of the backward variable selection (i.e. variables at the bottom of the table were deleted first from the full model). Variables retained in the minimal model are shown in bolditalics on top. numDF specifies the number of degrees of freedom for each variable. The difference in Akaike’s Information Criterion (ΔAIC) is shown for the variables that were deleted from the model. Both the *p* value from the likelihood ratio test (p-value) and the ΔAIC values were considered and variables were only retained in the model if their deletion resulted in a significant p-value and a ΔAIC > 2. In the minimal model, fixed factors alone explained 66% of the deviance (R^2^m = 0.66), while both fixed and random increased the deviance explained by 4% (R^2^c = 0.70)

**Fig. 2:**
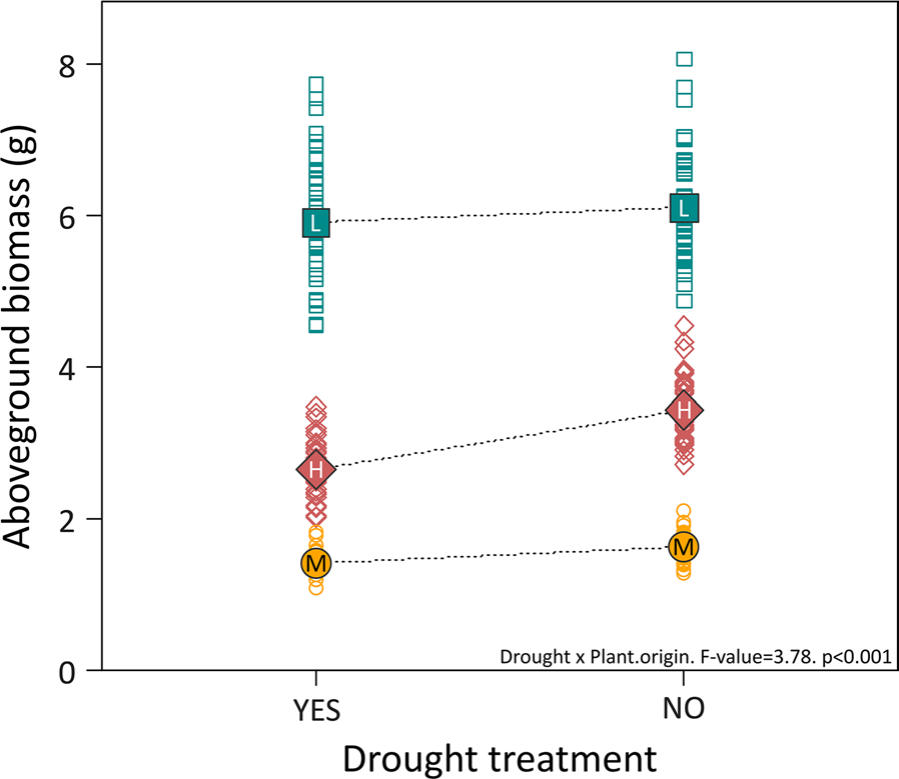
Effect of drought on aboveground biomass for each provenance. Open symbols are model predictions after standardizing by initial size. Mean values are displayed as filled symbols with letter code per region as in Fig. 1. F-value and significance of the interaction are displayed

Drought reduced the growth of each provenance differently (drought by plant origin interaction, Table 2, Fig. 2). Provenances from high elevations were most affected by the stressful conditions, while seedlings from middle and low elevation regions barely responded to the treatment, independent of the soil in which they were growing. This may suggest pre-adaptation to increasingly stressful conditions in our middle to low elevations, while populations at higher altitudes seemed less adapted to drought as they normally experience colder and wetter climate conditions (Table 1).

**Fig. 3:**
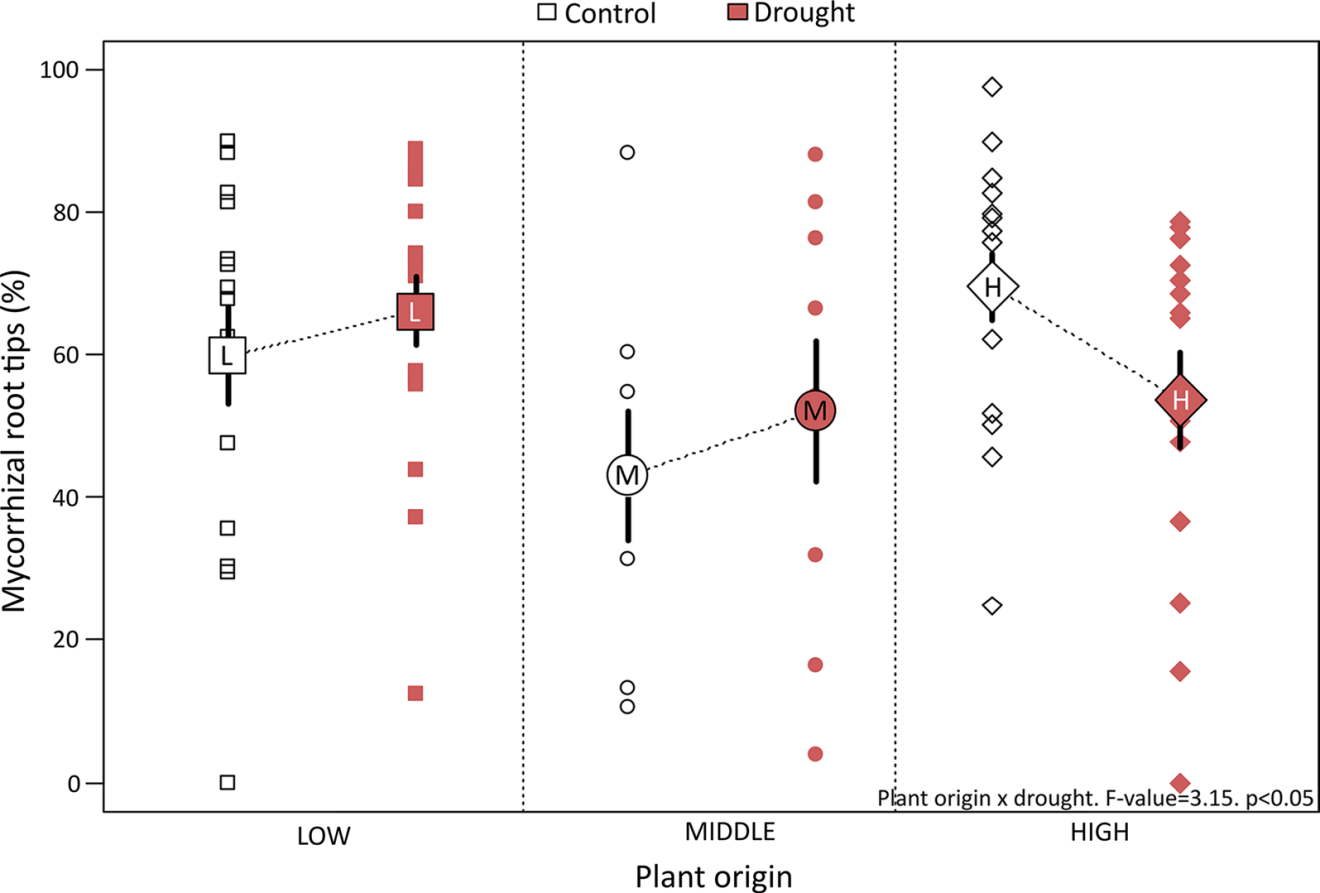
Effects of drought on the percentage of mycorrhizal root tips. Small symbols denote raw measurements while mean values per provenance are displayed as big symbols with standard error bars. F-value and significance of the interaction are also displayed

Two interactions significantly affected the rate of mycorrhizal colonization: those between drought and plant origin, and between soil origin and inoculum (Table 3). Regarding the first interaction, for two of three provenances, we observed an increase in the number of mycorrhizal root tips in the seedlings subjected to drought (Fig. 3), supporting a role for mycorrhizal interactions in plant resistance to extreme events. Consistent with this idea, seedlings from higher elevations, which were the most affected by drought (Fig. 2), also had reduced mycorrhizal colonisation under stressful conditions (Fig. 3). In contrast, inoculation with active soil significantly increased the amount of mycorrhizal root tips for the higher and lower elevation provenances, most strongly for the higher elevation seedlings (Additional file 1: Fig. S2).

**Table 3:** Model selection and levels of significance in the generalized linear model analysing the percentage of mycorrhizal root tips

Mycorrhizal root tips (%)				
Explanatory variable	numDF	F-value	p-value	
***Soil.origin***	2	0.18	0.84	–
***Plant.origin***	2	2.26	0.11	–
***Drought***	1	0.28	0.60	–
***Inoculum***	1	1.82	0.18	–
***Block***	1	1.47	0.23	–
***Drought: plant.origin***	2	3.15	0.04	*
***Soil.origin: inoculum***	2	3.54	0.03	*
Drought: inoculum	1		0.09	n.s.
Plant.origin: inoculum	2		0.21	n.s.
Drought: soil.origin	2		0.30	n.s.
Soil.origin: plant origin	4		0.73	n.s.
Drought: plant.origin : inoculum	2		0.05	n.s.
Soil.origin: plant.origin : inoculum	4		0.11	n.s.
Drought: soil.origin : plant.origin	4		0.11	n.s.
Drought: soil origin : inoculum	2		0.49	n.s.
Drought: soil.origin : plant.origin : inoculum	4		0.09	n.s.

Variables are shown in reverse order of the backward variable selection (i.e. variables at the bottom of the table were deleted first from the full model). Variables retained in the minimal model are shown in bolditalics on top. numDF specifies the number of degrees of freedom of each variable

## Discussion

### No evidence for local adaptation to soil

Our results show that plant and soil origins interact to affect the performance of transplanted seedlings. However, we did not observe an advantage for seedlings grown with home soil, as would be expected in the case of tree local adaptation. Instead, two of the three studied provenances produced less aboveground biomass when transplanted into pots inoculated with local soil, which might suggest maladaptation of *Fagus* to local soil biota, such as specialized antagonists [48]. It is also possible for ectomycorrhizae to behave antagonistically [10], although it is unclear what determines whether the fungi act as mutualists or parasites. In the case of parasites and pathogens, host maladaptation could be caused by a higher ability of the antagonists to adapt to their hosts than of the hosts to adapt to the antagonists, thanks to their bigger population size and shorter generation time [49, 50]. In these ‘Red Queen dynamics’, the parasites are ahead in the co-evolutionary arms race [16] and, as a consequence, plant performance can be increased by physically escaping from highly adapted antagonists, through dispersal, somewhat analogous to the ‘enemy release’ hypothesis that leads to greater performance of invasive species in their introduced range [51]. Although our results are consistent with antagonist driven host maladaptation, we did not find a difference between inoculated and non-inoculated treatments in the degree of local disadvantage, as would be expected if maladaptation was biotically driven. This is not because the inoculation effects had disappeared because even at the end of the experiment inoculated soil had significantly more mycorrhizae than non-inoculated soils. It is therefore unclear what caused the signal of soil maladaptation even in the non-inoculated pots. Maladaptation to abiotic soil conditions would be more surprising but could explain our results. Alternatively, the different abiotic soil conditions may have selected for different soil communities as biota recolonised the pots. To determine with certainty which of these hypotheses is more likely, characterisation of the soil communities of the different provenances would be needed. Nevertheless our results do show that variation in soils is an important driver of tree seedling performance and that effects depend on the genetic origin of the seedlings.

It should be noted, however, that due to our experimental design, there are some caveats that should be taken into consideration when interpreting our results. First, despite washing their roots, seedlings might have started the experiment with different rhizosphere communities and therefore with their own mycorrhizae and pathogens present. Given that the soil had different effects it is unlikely that this could explain all the results, however, initial differences between seedlings could have affected the soil biota that established in each pot. Similarly, there was no opportunity for us to ascertain the degree of contamination by soil biota from the surrounding of the experiment. This is a common problem for all soil experiments done under open environmental conditions (in contrast to closely controlled environments, e.g. an ECOTRON). Finally, since we could not reliably measure the mycorrhizal community composition for each population, we cannot ensure that the inoculum was effective in creating different mycorrhizal communities in our treatments. Previous literature (discussed below in each section) supports our methods, but these shortcomings should be taken into account.

### Provenance differentiation

Contrary to our expectations, we observed clear and consistent differences between our tree provenances. In widespread and highly connected populations, genetic differences between populations are commonly assumed to be low [52], while within-population variability remains high [30]. Therefore, for widespread species, only populations located at the edges of their natural range are expected to show strong genetic differentiation [28]. However, our results indicate that seedlings transplanted from populations in the central part of a species range can still be strongly differentiated despite presumably high levels of gene flow between the populations. It is possible that the provenances differed in their response to the growing conditions in Bern. These were most similar to the conditions in the origin of the higher elevation population, in terms of precipitation, but were warmer than conditions experienced by any population in their origin, and actually closer to the temperatures commonly experienced in the lower elevation region. It is therefore not clear that climatic conditions would have favoured the growth of any particular provenance. It remains unclear what factors might be driving this population differentiation and further work with these provenances using both reciprocal transplant experiments and common garden experiments are needed to identify the drivers of divergent selection.

Our results also show significant differences between provenances in their ability to tolerate drought. Climate change models currently do not consider local adaptation to drought or extreme climate events, but if adaptation to local, stressful conditions is widespread, this could modify the responses of forests to the changing climate. It would mean that the effect of drought on forests is likely to be highly variable [18]. Intraspecific differences in drought tolerance is a key component of future local adaptation that needs to be considered both in climate change models [53], and in the development of local adaptation theory [54].

An important factor to consider in experimental tests of local adaptation are maternal and storage effects, which can confound the observed adaptation patterns [55]. Despite their relevance, few studies explicitly control for them [4]. Usually, plants are grown for 1- 2 generations under common conditions to control for maternal and storage effects, and only the subsequent generations are used in the transplants (e.g. [55]). However, this methodology cannot be applied to long-lived organisms, such as trees, for obvious practical reasons. Therefore, alternative methodologies have been proposed. We used one of the most common ones, which accounts for maternal and storage effects by standardizing growth by initial plant size (e.g. [38]). This aims to control for differences in levels of stored reserves and in pre-experimental conditions between the different provenances. Using naturally regenerated plants allowed us to work with older seedlings and to explore the effects of our treatments on seedlings which have already passed some early environmental filters. It would interesting, however, to compare the results from seedlings transplanted from naturally regenerated trees, as in our case, with plants grown from seeds or under sterile conditions, to compare and confirm the strength of the observed patterns, and observe any potential confounding effects of transplanting established field seedlings into greenhouse pots. Unlike plants germinated under experimental conditions in isolated pots, naturally regenerated plants can profit or be harmed by the extensive mycorrhizal network that connects tree roots in natural forest soils. This is likely to affect the ability of fungi to uptake and transport water and thus, potentially the plant’s drought resistance. We cannot explore this question using our experimental design, but future work that combines plants germinated under experimental conditions and naturally regenerated ones would be useful to test the importance of these factors.

### Mycorrhizal abundance

Contrary to what was has been observed for other species [10, 36], we did not find an overall increase in mycorrhizal colonisation when tree seedlings were grown on their local soil (soil origin by plant origin interaction, Table 3). We did, however, observe an effect of inoculation for two of the provenances, which suggests that inoculation did affect the mycorrhizal community. Overall, our results rather suggest that mycorrhizal communities were unresponsive to plant origin, soil origin, and stressful conditions; and indicate that these associations may be more flexible than usually considered. However, even without changes in abundance, mycorrhizal species composition could have differed between provenances. It is also possible that the seedlings maintained part of the associations they already had despite our efforts to carefully clear the roots before transplanting, or that particular associations with symbionts present in the experimental site happened to favour a particular tree phenotype [21]. We also cannot exclude the possibility that a provenance or particular community of inoculum is, by chance, better adapted to grow under our experimental conditions. This adaptation to laboratory conditions is a shortcoming of all common garden approaches (discussed in [54]). We have no expectation for any of our provenances or soil communities to be better adapted to growing on our potting soil mix, since none of the soils in the origin of the provenances are located in peatlands or flooded areas, which would be closer to the potting soil. However, a better assessment of the fungal community composition and parallel reciprocal transplant experiments would be necessary to clarify this in the future.

The small differences in mycorrhizal colonization between local and foreign soils might also suggest that mycorrhizal communities do not differ between regions as much as expected, likely due to the high connectivity between *Fagus* populations in central Europe [29], which would tend to homogenise the populations of associated biota. This is in agreement with recent studies that have shown very low levels of endemism in arbuscular mycorrhizae, although these may differ in their response compared to ectomycorrhizae [56]. If ectomycorrhizae also show low endemism then trees would be unlikely to adapt to local mycorrhizal communities. On the other hand, Pena et al. [57] recently showed that mycorrhizal communities occurring in different forest types in the Biodiversity Exploratories were highly differentiated. Since our seedlings were sampled from monocultural *Fagus* stands in all three regions, it is possible that we would have found larger differences in mycorrhizal communities if we had compared between forest types rather than geographical locations.

The small differences we observe in mycorrhizal colonization between provenances (see e.g. [58]) likely indicate that some community homogenization have happened during the common garden experiment, despite our attempts to minimize cross-contamination between plots. This colonization via greenhouse borne spores or between pots is difficult to quantify and prevent, and it can potentially confound the measurements of mycorrhizal diversity and composition. And it is possible that a larger ratio of inoculum/substrate would have provided better resistance to this homogenization or potential external colonization by common mycorrhiza (but see [36]). Previous experiments that used the same substrate as we employed, suggested it is relatively resistant to external mycorrhizal colonization, as non-inoculated plants remained free of root colonization (in their case of arbuscular mycorrhizal fungi) throughout the experiment [59]. However, the role of specialized mycorrhizae might be underestimated in our experimental settings compared with that of natural conditions. While pre-grown sterile seedlings would have reduced the problem of potential pre-existing spores or fungi over the roots, they would have been similarly sensitive to cross-contamination between pots. However, by using naturally established plants we are able to work with older, more mature seedlings, and therefore to test for local adaptation at a later life history stage than is common in tree seedling experiments.

### Effects of drought on the plant-mycorrhizae association

We found a small increase in mycorrhizal colonization under more stressful environments, consistent with the hypothesis that plant resistance to extreme events is affected by mycorrhizal associations [60, 61]. This could be a result of mycorrhizae increasing host nutrient uptake [62] which may in turn facilitate drought resistance. These small differences appeared even under the light drought treatment which we imposed here and under more extreme droughts, a stronger response of mycorrhizae could be expected.

Drought resistance is likely to be of key importance for seedling survival under future climatic conditions [53] and its dependence on specialized, small-scale biotic interactions may imply that tree responses to future conditions can vary substantially depending on whether climate change decouples interactions with their soil biota [23, 63]. An increasing number of studies support this idea: for example, Liang et al. [64] showed that altitudinal migration of trees in response to climate change can be strongly modified by the biotic interactions that plants encounter in the new area. Similarly, forest stands experiencing recent high mortality had lower colonization rates by ectomycorrhizae, suggesting that the reduced mycorrhizal associations had hampered tree performance and increased the likelihood of tree mortality in the face of extreme conditions [65]. While models predicting species responses to climate change have increased in complexity and accuracy by incorporating some biotic and abiotic interactions [62, 66], and intra-specific variability [53], the interactions between plant and soil biota are yet to be included.

## Conclusion

Soil effects on seedling performance suggested a general dominance of antagonistic interactions between soil biota and *Fagus sylvatica* provenances in Germany. However, the effect of soil origin was smaller than that of plant origin. We found a consistent hierarchy in population performance, with lower elevation provenances showing increased growth compared with medium and higher elevation ones. This differentiation in seedling performance was not related to our experimental variables. We did, however, find evidence for a positive effect of mycorrhizal associations on seedling resistance to stressful conditions. Overall, our results suggest that the genetic differentiation between populations of *Fagus sylvatica* and their interactions with generalist soil biota are most important for seedling responses to climate change.

## Additional file

**Additional file 1: Table S1.** Initial heights and diameter measurements. **Table S2.** pH of the provenances’ original soil before and after sterilization, and substrate used. **Table S3.** biomass increment for all treatment combinations. **Fig. S1.** Soil chemical characteristics of the provenances’ original soil, and of the substrate used. **Fig. S2.** Effect of inoculation on the percentage of mycorrhizal root tips per soil origins.

## Abbreviations

AGB: aboveground biomass
AIC: Akaike’s information criterion
*Fagus*: *Fagus sylvatica*
glm: generalized linear models
lme: linear mixed models
Mycorperc: percentage of mycorrhizal colonization
