# Supplementary material for: Fagus sylvatica seedlings show provenance differentiation rather than adaptation to soil in a transplant experiment
Source: BMC Ecol. 2018 Oct 3;18:42. doi: 10.1186/s12898-018-0197-5 (PMC6171197; doi:10.1186/s12898-018-0197-5)
Supplement: Supplementary file 1 — Additional file 1: Table S1. Initial heights and diameter measurements. Table S2. pH of the provenances’ original soil before and after sterilization, and substrate used. Table S3. biomass increment for all treatment combinations. Fig. S1. Soil chemical characteristics of the provenances’ original soil, and of the substrate used. Fig. S2. Effect of inoculation on the percentage of mycorrhizal root tips per soil origins. [file 12898_2018_197_MOESM1_ESM.docx]

**Appendix 1**

**Table S1** | Initial height and diameter for the analysed provenances pre-experiment

|  |  | *Height (cm)* | *Diameter (mm)* |
| --- | --- | --- | --- |
| Low | Pre-establishment | 21.97 ± 0.50 | 4.55 ± 0.10 |
|  | Post-establishment | 21.92 ± 0.51 | 5.10 ± 0.11 |
| Middle | Pre-establishment | 44.71 ± 1.03 | 5.09 ± 0.10 |
|  | Post-establishment | 41.42 ± 1.95 | 4.35 ± 0.18 |
| High | Pre-establishment | 18.52 ± 0.34 | 3.01 ± 0.06 |
|  | Post-establishment | 18.46 ± 0.36 | 3.05 ± 0.06 |

**Table S2** | pH values for each of the provenances’ original soil before and after sterilization treatment, as well as for the standard substrate used as growing medium.

| *Soil* | *Sterilized* | *pH* |
| --- | --- | --- |
| Low | Yes | 5.23 |
|  | No | 5.02 |
| Middle | Yes | 7.23 |
|  | No | 7.51 |
| High | Yes | 5.12 |
|  | No | 4.49 |
| Standard substrate | Yes | 5.86 |

**Table S3** | Mean biomass increment for all combinations of plant and soil origin used in our experiment. The standard deviation (sd), and standard error (se) is also displayed. Shown values are model predictions after correcting for initial size differences.

| *Plant origin* | *Soil origin* | *Mean biomass increment (g)* | *sd* | *se* |
| --- | --- | --- | --- | --- |
| M | **L** | 1.52 | 0.20 | 0.040 |
| N | **L** | 5.53 | 0.53 | 0.077 |
| H | **L** | 3.47 | 0.56 | 0.081 |
| M | **M** | 1.67 | 0.19 | 0.040 |
| L | **M** | 5.92 | 0.55 | 0.081 |
| H | **M** | 2.81 | 0.43 | 0.063 |
| M | **H** | 1.41 | 0.15 | 0.039 |
| N | **H** | 6.75 | 0.63 | 0.093 |
| H | **H** | 2.98 | 0.47 | 0.074 |

**
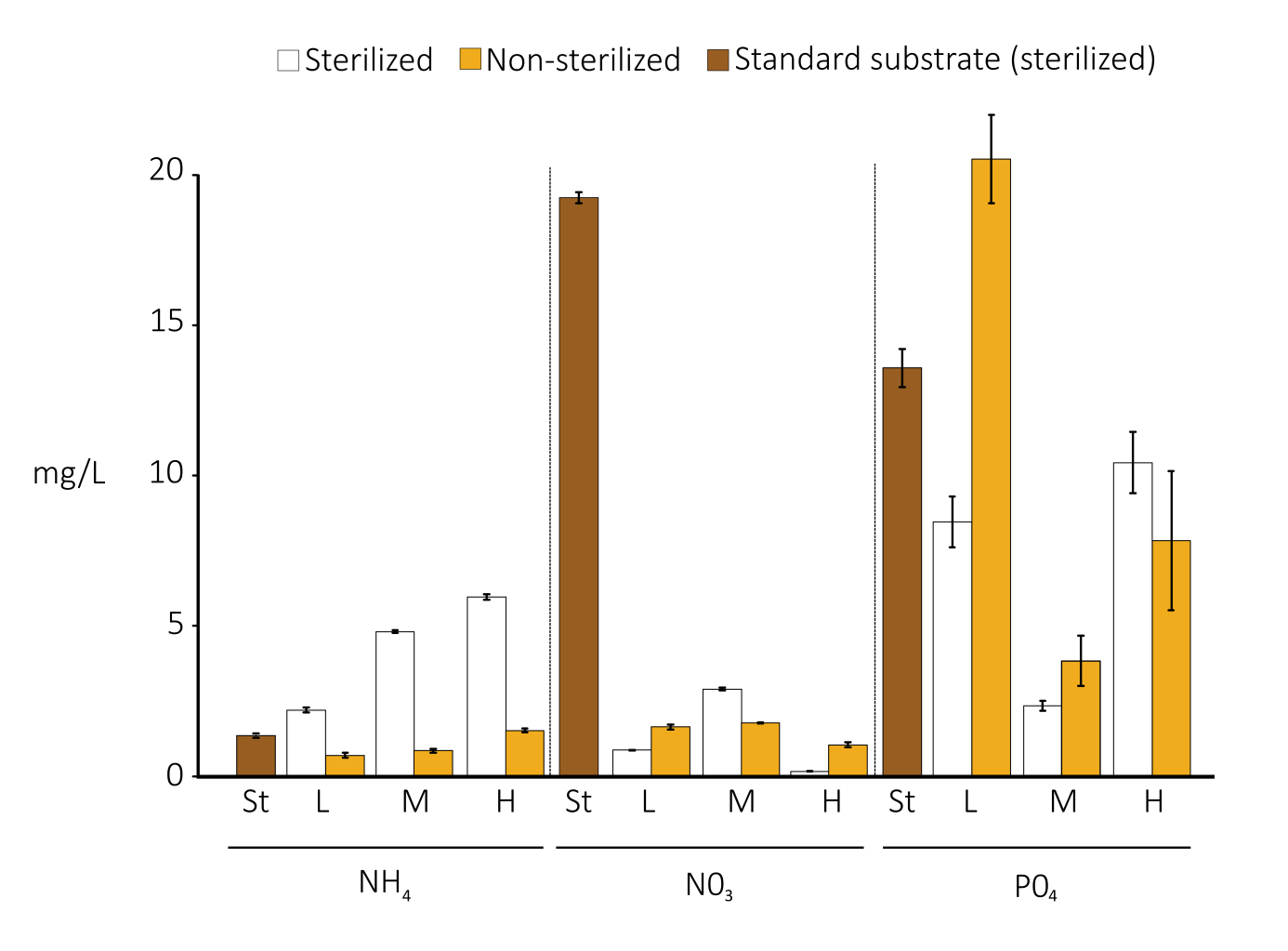
**

**Fig. S1| Soil chemical characteristics for the low (L), middle (M), high (H) elevation provenances, and the standard sterilized substrate used as growing medium (St).** The concentration of NO_3_ in the standard substrate is remarkable (see details in Methods). Note that Nitrogen measurements are calculated by dry combustion using ‘CN Elemental Analyzer’ and thus represent the combination of organic and inorganic nitrogen in the soil.

**
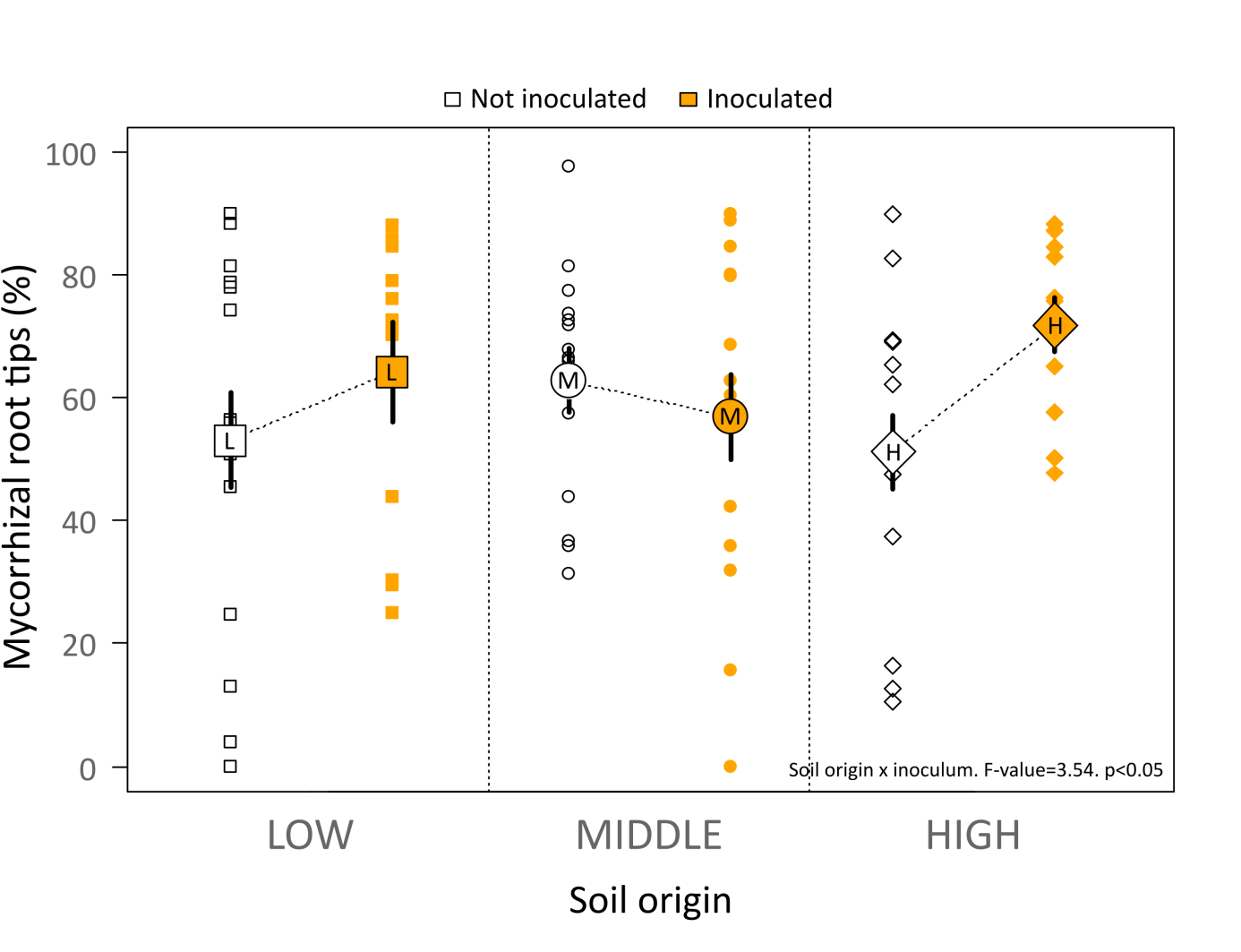
****Fig. S2| Effect of inoculation on the percentage of mycorrhizal root tips per soil origins.** Small symbols denote raw measurements while mean values per provenance are displayed as big symbols with standard error bars. Letter code per region as in the X-axis and Table 1. F-value and significance of the interaction are also displayed.
